# Active video games to promote physical activity in children with cancer: a randomized clinical trial with follow-up

**DOI:** 10.1186/1471-2431-14-94

**Published:** 2014-04-05

**Authors:** Lotta Kauhanen, Liisa Järvelä, Päivi M Lähteenmäki, Mikko Arola, Olli J Heinonen, Anna Axelin, Johan Lilius, Tero Vahlberg, Sanna Salanterä

**Affiliations:** 1 Department of Nursing Science, University of Turku, Lemminkäisenkatu 1, FI-20014 Turku, Finland; 2 Department of Pediatrics, Turku University Hospital, Kiinamyllynkatu 4-8, 20520 Turku, Finland; 3 Hospital District of Southwest Finland, Kiinamyllynkatu 4-8, 20520 Turku, Finland; 4 Department of Pediatrics, Tampere University Hospital, PL 2000 Tampere, Finland; 5 Paavo Nurmi Centre, Sports & Exercise Medicine Unit & Department of Health and Physical Activity, University of Turku, Kiinamyllynkatu 10, 20520 Turku, Finland; 6 Department of Information Technologies, Åbo Akademi University, Joukahaisenkatu 3-5A, 20520 Turku, Finland; 7 Department of Biostatistics, University of Turku, Lemminkäisenkatu 1, FI-20014 Turku, Finland

**Keywords:** Pediatrics, Cancer, Physical activity, Sedentary behavior, Motor performance, Fatigue, Rehabilitation, Active video games

## Abstract

**Background:**

Low levels of physical activity, musculoskeletal morbidity and weight gain are commonly reported problems in children with cancer. Intensive medical treatment and a decline in physical activity may also result in reduced motor performance. Therefore, simple and inexpensive ways to promote physical activity and exercise are becoming an increasingly important part of children’s cancer treatment.

**Methods:**

The aim of this study is to evaluate the effect of active video games in promotion of physical activity in children with cancer. The research is conducted as a parallel randomized clinical trial with follow-up. Patients between 3 and 16 years old, diagnosed with cancer and treated with vincristine in two specialized medical centers are asked to participate. Based on statistical estimates, the target enrollment is 40 patients. The intervention includes playing elective active video games and, in addition, education and consultations for the family. The control group will receive a general recommendation for physical activity for 30 minutes per day. The main outcomes are the amount of physical activity and sedentary behavior. Other outcomes include motor performance, fatigue and metabolic risk factors. The outcomes are examined with questionnaires, diaries, physical examinations and blood tests at baseline and at 2, 6, 12 and 30 months after the baseline. Additionally, the children’s perceptions of the most enjoyable activation methods are explored through an interview at 2 months.

**Discussion:**

This trial will help to answer the question of whether playing active video games is beneficial for children with cancer. It will also provide further reasoning for physical activity promotion and training of motor skills during treatment.

**Trial registration:**

ClinicalTrials.gov identifier: NCT01748058 (October 15, 2012).

## Background

The risk of any child developing pediatric cancer is 1–2/10,000, and about one-third of these cases are leukemias [1]. Because of the improvements in treatments, the overall survival rate has increased, and 5 years after diagnosis over 80% of the diseased children are still alive.

Previous studies indicate that children with cancer gain weight during their treatment and have difficulties in returning to their normal weight after treatment [2]. Younger patients in particular start to gain weight as early as 6–12 months after diagnosis and usually continue to gain weight for one year after their treatment [3-6]. Low physical activity and being overweight may lead to an increased risk of chronic diseases, such as metabolic syndrome, type two diabetes and cardiovascular diseases [7]. Children with cancer are also at increased risk of osteoporosis [8]. Furthermore, long-term survivors of childhood cancer, especially leukemia, have been reported to have problems with bone mineral density [9] and lower fitness than age mates [10]. Good physical fitness is probably the most important factor in achieving a normal bone mass in childhood leukemia survivors [11].

Cancer treatments such as vinca-alkaloids may cause peripheral motor or sensory neuropathy, leading to clumsiness, muscular weakness and deficient motor skills. Children with cancer may also suffer from reduced muscle elasticity and ligament laxity [12-15]. Peripheral neuropathy, muscle weakness and mobility deficits may also continue years after treatment [16,17]. Normal participation in physical activity, school physical education classes and group activities are interrupted at a time when the child’s motor skill development is rapid and highly important [18]. The child’s protected condition and an increased risk of infection result in a secluded life. Thus, when physical activity decreases, due to many factors during treatment [19], and the disease and the treatment’s side effects affect the child, his/her motor performance and motor skill development usually lags behind that of healthy peers. Furthermore, physical fitness is lower in acute lymphoblastic leukemia (ALL) survivors than in their healthy peers [20].

Most studies reporting physical activity levels in children with cancer have investigated the phenomenon in the maintenance phase or years after treatment; only a few studies have focused on physical activity levels during early treatment [21,22]. It is known that during the first year of treatment children with cancer perform significantly less moderate-to-vigorous physical activity than healthy controls, indicating that these children remain mostly inactive during the initial year of treatment even though the recommendations encourage age-appropriate physical activity that is also individually appropriate [23]. Physical activity recommendations for children with ALL indicate that at the maintenance phase of treatment they should progress to an activity level close to the recommendations set for healthy children [24]. During the induction and consolidation phase, light physical activity according to the individual’s condition is recommended. [24]. In a recent randomized trial [25] in which the recruitment was conducted at the time of diagnosis and the intervention group received exercise program at the beginning of the cancer treatment motor performance and passive ankle dorsiflexion decreased equally in both groups. Also the body fat increased similarly in both groups. Hartman et al. [25] suggest that this was due to unsatisfactory commitment to the exercise program. Therefore it is important to develop motivating exercise programs.

Physical activity and different exercise programs during cancer therapy have been approved as beneficial in improving children’s cardiovascular fitness, muscle strength, motor performance and flexibility and in reducing fatigue [26]. Physical activity and exercise interventions have been found to be particularly effective when carried out in supervised settings like in hospital environment [19,27,28]. However, the evidence is still limited due to small sample sizes and lack of randomization in most trials. Therefore, it is necessary to develop and produce evidence of safe, effective exercise and motivating interventions for children with cancer.

Active video games, such as Nintendo Wii™ (Nintendo Co., Ltd., Kyoto, Japan) Xbox Kinect (Microsoft Co., Redmond, WA, USA) and Konami Dance Dance Revolution (Harmonix Music Systems Inc., Cambridge, Massachusetts, USA), have become amazingly popular among children and adolescents. For instance, the market best seller, Nintendo Wii™, sold 99 million games consoles in 2012 worldwide [29]. An active video game is one in which playing the game is based on the player’s movements. Thus, these games may provide one possibility for decreasing sedentary screen time and motivating children to be more physically active. The explosive popularity of these games has also inspired physiotherapists and other health professionals to use and study them in rehabilitation [30-32]. Even though these games may not be the solution to achieving the recommended physical activity in healthy children, they might be helpful in breaking up sedentary time in some special circumstances, such as during hospitalization or isolation. These games are also feasible for cancer patients because they can be played in patient rooms, where the risk of infection is minimized. In addition, children are motivated to play them, as they are safe and fun to play [33].

Evidence for and against active video gaming to promote physical activity exists, but most studies concerning active video games used for rehabilitative purposes have produced positive results [30]. Playing active video games in laboratory circumstances raises healthy children’s energy consumption and heart rate. Active video-game play equals light-to-moderate physical activity [31,32], but in more naturalistic settings, like at home, the results have not been as promising [34]. Baranowski et al. [34] suggest this to be due to unsupervised settings where players did not reach the needed intensity or compensated the increased physical activity by being less active at other times of the day [34]. However, in rehabilitation and restricted circumstances, such as during hospital stays, these games may have benefits. In healthy population, active video games may not be an alternative to being physically active but can be useful in populations with limitations, as these games can be played even when being seated. A review by Primack et al. showed that active interventions based on video games improved 59% of the physical therapy outcomes, 50% of the physical activity outcomes and 42% of pain distraction outcomes [30]. Nevertheless it should be noticed that the quality of the studies concerning active video games has mostly been poor [30].

Active video games have been studied also in children with cerebral palsy [35,36], developmental delay [37] or disability [38], acquired brain injury [39] and lower limb amputations [40] with promising results [30] but, to the best of our knowledge, active video games have not yet been studied in children with cancer alone. However, there is a protocol published in which active video gaming is used for exercise purposes in pediatric autologous stem cell transplant patients [41].

### Study aims

The primary aim of this study is to evaluate the efficacy of active video games with regard to the promotion of physical activity in children with cancer. The ultimate goal is to motivate children with cancer and their families to integrate active lifestyle choices into their everyday life, even during treatment, to decrease the negative impact of high levels of sedentary behavior. The results will help answer the question of whether playing active video games has a positive effect on the health of children with cancer.

Our hypothesis is that the possibility to play active video games and guidance given to the family about physical activity during cancer treatment promote children’s physical activity and shorten sedentary periods when compared to the control group, who receive only a general recommendation to be active.

The detailed research questions and hypotheses of the study are:

1. Is playing active video games effective in increasing the physical activity of children with cancer during cancer treatment?

a) It is hypothesized that the possibility of playing active video games during cancer treatment promotes children’s physical activity compared to controls.

2. Is playing active video games effective in promoting motor performance during cancer treatment?

b) It is hypothesized that children’s motor performance in the intervention group is maintained better than in the control group.

3. Is playing active video games effective in reducing experienced fatigue by children with cancer?

c) It is hypothesized that the children in the intervention group experience less fatigue during cancer treatment than the children in the control group.

4. Do playing active video games have impact on children’s body mass index and the development of metabolic risk factors in children with cancer?

d) It is hypothesized that playing active video games has a positive impact on children’s body mass index and metabolic risk factors during cancer treatment.

5. What are the children’s and families’ perceptions of the most enjoyed activation methods during cancer treatment?

The protocol follows the methodological guidelines for clinical trials outlined in the CONSORT statement [42].

## Methods

### Trial design

This research is conducted as a parallel randomized clinical trial with follow-up. The study subjects are randomly allocated into intervention and control groups.

### Participants

The study population is children and adolescents diagnosed with ALL or other cancer outside the central nervous system (e.g. Hodgkin and non-Hodgkin Lymphomas, neuroblastoma, Wilms tumor, rhabdomyosarcoma, retinoblastoma and Ewing sarcoma). The treatment for different forms of cancer varies considerably, however; one inclusion criteria is that the treatment regimen has to include vincristine. The treatment duration and dose is taken into account in the data analyses. Patients with central nervous system tumors are not included as these tumors may cause severe and more permanent motor performance difficulties complicating the study design. In addition, these conditions may require brain surgery and the physical activity and exercise recommendations may have great individual differences, even though these patients would also benefit from physical activity.

The eligibility criteria to this study are: 1) the patients are aged 3–16 years at the time of recruitment, 2) the treatment regimen includes vincristine and 3) the patient is treated in either of the designated hospitals (Turku University Hospital or Tampere University Hospital, Finland). The lower age limit of 3 years for the first inclusion criterion is chosen because in the included group 3-year-old children are estimated to be the youngest that may have the competence to learn to play the easiest active video games of Wii™ Fit (Nintendo Co., Ltd., Kyoto, Japan). Also the motor performance measure Movement ABC-2 (MABC-2, 2nd Edition, Pearson Education, Ltd., London, UK) is designed to measure 3- to 16-year-old children’s motor performance [43]. The second inclusion criterion is chosen because the sample is aimed to be as comparable and homogeneous as reasonably possible and because the homogeneity in this sample varies in many other ways such as age and diagnosis. In addition, vincristine treatment may cause peripheral neuropathy increasing the need of physical activity and exercise. The sampling method used in these two university hospitals is simple random sampling—all new patients meeting the eligibility criteria are given the possibility to be included. Together, these two hospitals treat approximately 40% of the pediatric cancer patients in Finland, which accounts for 20–30 new cases eligible for this study annually. The participants are excluded if they have other diseases limiting their physical or cognitive function, epilepsy, or they are not able to communicate in Finnish, Swedish or English. The target sample size is calculated from the main outcome, using a power analysis.

### Recruitment

Children are asked to participate within a week of their primary cancer diagnosis, or as soon as possible after that. The attending physician verifies the children’s eligibility for the study and contacts the researcher. Eligible children and their caregivers receive written and oral age-appropriate research information from the researcher, and informed consent is obtained the next day at the earliest. The family may consider, for as long as necessary, whether to participate. The children need to give their consent at least verbally and parents sign the written informed consents. In addition, children who have already learned to write their name sign their own written consents. According to Finnish legislation, participants over 15 years old may individually decide whether they are willing to participate [44]. The recruitment will continue until the target sample size is achieved.

### Intervention

The physical activity in the intervention is based on active video gaming. The intervention consists of playing elective active Nintendo Wii™ games daily, for at least 30 minutes, during hospitalization and at home for 8 weeks in total, with consideration of the participants’ individual conditions. Physical activity is not allowed during fever and vomiting or nausea episodes, or if the medical condition changes acutely. The physical activity intensity is recommended as light-to-moderate. One way to monitor the relative intensity of activity is to monitor the respiration and breathing rate during the activity [45]. The parents are guided that light-to-moderate intensity physical activity equals only to slightly increased breathing rate, still feeling “easy to breath” or “slight breathlessness” and they are advised to monitor the child’s condition before and during the physical activities. At the beginning of the study, the attending physician verifies the children’s ability to physical activity, and, if there are significant changes to the child’s condition, the researcher and the physical therapists who conduct the motor performance testing are informed. The children receiving anthracycline therapy are also followed by a cardiologist and regular echocardiograms.

The intervention includes information and recommendations for physical activity and guidance on which Nintendo Wii™ Fit games to play and how to play them. All children in the intervention group will receive the same written instructions and individual face-to-face teaching, where examples about suitable games for each age group are presented. For 3- to 6-year-old children, games such as Hola hoop or Jogging are recommended based on the easiest difficulty levels. For children aged 7- to 10-years, the recommended games include such as Island Cycling, Rhythm Kung-Fu, Hola hoop and Jogging and for 11- to 16-year-old children there are recommended games from all the four types of exercises (aerobics, balance, strength training, yoga). This guidance and instruction are given by a physiotherapist in the hospital. The physiotherapist contacts the participants in the intervention group via telephone for consultation during the intervention in order to encourage children to play and be active. Children in the intervention group have the game console and games at home for the duration of the intervention period.

Some limitations of the intervention must be mentioned: children may learn to play the games with incorrect execution of movements, which decreases the amount of activity while playing. However, the physical therapist teaches the games to the intervention group as they should be played, and parents are guided to watch that the children play them correctly. It is also more difficult to cheat the balance board used in the intervention than the remote control, and even if the child is executing a movement incorrectly, playing the game still needs advanced coordination with the timing of the movements. The second concern about the intervention is that how well the smallest enrolled children can experience success when playing the game. Too difficult tasks may promote boredom and unsatisfactory commitment to the program. This might be the case among the very youngest children enrolled to the study. However, our experiences have been positive so far. After this study we get valuable directions to develop the intervention further and even new easier activity promoting video games might have come to the market.

If the children or guardians have questions about the intervention or physical activity recommendations they are guided to contact the ward or the research group. If the question is about the child’s health, they are guided to contact the ward staff. Both study wards and their staff are well informed about the ongoing study and will let the researcher know of any questions concerning the study or its procedures.

In this study, the control group receives only general advice for physical activity for 30 minutes per day. They do not receive guidance on playing active video games or telephone consultations after being discharged from the first medical treatment period. The controls have the normal treatment so that if they need physical therapy referral or consultation from a physical therapist they will receive the normal treatment similarly as the children in the intervention group. All the physical therapy session documentation is collected in the end of the study and will be taken into consideration when analyzing and reporting the data.

### Outcome measurements and time-points of assessments

The main outcomes*—physical activity* and *sedentary behavior*—*,* are measured with four different outcome measures. The main outcome measurement device for physical activity is a three-dimensional accelerometer, The Fitbit Tracker (Fitbit, Inc., San Francisco, CA). An accelerometer is a motion sensor which detects changes in movement. An accelerometer provides a valid and reliable assessment about achieving objective data of children’s physical activity [46]. We have not found a published validation study about the Fitbit Tracker (Fitbit, Inc., San Francisco, CA) so far but our research group will conduct a validation study before the data analysis of this study to fulfill this limitation. The accelerometer is used at 1^st^ week of the intervention and at 1 year. The device is worn for one week at a time so that the measurement includes both week and weekend days. It is worn on the waist. Attaching accelerometer to waist it is relatively close to the body’s center of gravity and vertical movements are comparable to energy expenditure [47]. The accelerometer is not waterproof, so the study participants are guided to remove it while taking a shower or swimming. The times accelerometer is not worn are reported and given to the researcher when returning the accelerometer. Physical activity is also measured with two questionnaires and a self-assessment activity diary.

The first physical activity questionnaire assesses leisure-time physical activity in metabolic equivalent (MET) hours (METh) per week. The questionnaire contains multiple-choice questions about physical activity intensity, duration and frequency and it is widely used self-reported questionnaire [48]. The questionnaire has previously been used in studies of children’s and adolescents’ physical activity and vascular health [49-51]. The questionnaire correlated relatively well with the accelerometer data (r = 0.26–0.40) and pedometers (r = 0.30–0.39) [50]. For the children under 10 years old, this questionnaire was modified, the parents filling it in as a proxy report of their child. The other activity questionnaire was developed for this study and adds questions about the children’s physical activity and sedentary behavior, such as how much time the children spend in sedentary screen-based activities. The questions are divided into categories: sedentary behavior, light activities, moderate activities and vigorous intensity activities [52]. Report estimates the amount of these activities during a normal day.

The activity diary is completed for one week at every measurement point. The measurement points are: at baseline, 2 months, 6 months, 1 year and 2.5 years after the baseline. Using the activity diary data, we get self- and proxy-reported estimates about the frequency and duration of activity. Behaviors and activities are collected in 10-minute periods 24 hours a day, and the activities that are filled in the diary are divided into categories (sleeping, being awake but lying in bed, being awake and sitting, light activity, moderate activity, vigorous activity or playing active video games). Hence, the child’s acts are collected during the whole 24 hours for seven days in every measurement points. Moreover, the intervention group fills out a special game diary to separate the active videogame playtime and different games they play from other activity. The self- or proxy reported data from the diaries may not be precise but it will give directions and add valuable information to the objectively collected data.

According to literature, children under 10 years old may not be cognitively able to recall and report their physical activity reliably [53]. Therefore, 3- to 9-year-old children’s guardians fill out the questionnaires as a proxy report. Children aged 10 to16 years fill out the questionnaires independently. The questions have some age related modifications depending on whether the child or the guardian is designed to fill the questionnaire.

*Motor performance* is measured with the Movement Assessment Battery for Children (M-ABC) test [54,43]. The revised version M-ABC2 has organized the test age bands from 3 to 6, from 7 to 10 and from 11 to 16 years, so the test is suitable for the studied age group and it has age-related scores (3–16) [43]. The M-ABC2 has been established as a valid instrument for measuring children’s motor skills [55] and has been successfully used among cancer patients [56]. The M-ABC2 measures standardized tasks in three categories: manual dexterity skills, ball skills and balance skills [43]. The motor performance assessments are conducted during the inpatient stays or as a part of their normal treatment or control visits. If possible, the patients are not connected to any medical devices during the testing, and the testing is conducted in a therapy or test room that physical therapists use near the treatment ward. In some circumstances, if needed, the test may be conducted in the patient’s room. Depending on the child’s condition, some parts, i.e. jumping or balancing, might have to be skipped. All the modifications or skipped tasks and the reasons for those are documented carefully and considered during the data analyzes.

Children’s self-reported *fatigue* is reported by standardized PedsQL Multidimensional Fatigue Scale questionnaires. The PedsQL questionnaires have age-related scores, including the proxy report versions for parents [57]. The parents of the 3- to 4-year-old children fill in only the proxy report versions of the questionnaire. The 5- to 7-year-old children are helped to fill in the questionnaire, and their parents fill the proxy version. The 8- to 18-year-old children and their parents fill in the questionnaire independently. The PedsQL™ Multidimensional Fatigue Scale has shown fairly good internal consistency and responsiveness in measuring fatigue in children and adolescents with cancer [58].

To assess *the metabolic risk factors*, each participant’s height, weight, body mass index, resting blood pressure, waist circumference, and fasting blood sugar and insulin concentrations are measured at all measurement points. Invasive tests are carried out within the normal blood tests of the cancer treatment procedures so that no additional needle punctures into the patients are required. Metabolic risk factors are examined, since childhood cancer survivors have been shown to be more insulin resistant and have higher cardiovascular risks than healthy children [59,60].

Eight weeks after the first measurement point, each child, and guardian if the child is under 10 years old, are interviewed by the researcher (LK). This interview includes open questions about physical activity, and each child is asked about their perceptions of the most enjoyed activation methods during their cancer treatment in the hospital and at home. The aim is to find out about children’s own ideas on how they would become more active and what they would like to have in the hospital to inspire them to physical activity. The children in the intervention group are also asked about their experiences of active video gaming to gain a deeper understanding about the intervention. The interviews are voice recorded if the child and his/her family are comfortable with it and provide their consent. There are multiple aims for this qualitative section of the trial [61]. First, we want to identify the needs of this target population regarding how they would like to be motivated and activated and how that activation could manifest during periods of treatment and isolation. Second, it is useful to know the reasons for why some participants dropped out or did not participate. Third, we want the participants to describe the problems, if any, in the intervention for further research and clinical purposes. In addition, the interviews will help to evaluate and clarify the overall study findings [61].

**Figure 1:**
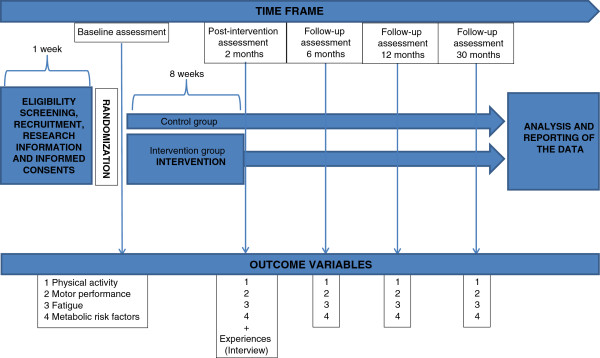
Outcome variables and time points of assessment.

The baseline demographics of all the participants include the time of diagnoses, type of cancer, treatment received and information of the disease status. This information is collected from the electronic health records. The time points of assessment and the outcome variables are described in Figure 1.

### Sample size calculation

To estimate the sample size requirements, the target sample size was defined by a power analysis using the primary outcome, physical activity. The calculation was made using the accelerometer counts, and the activity level baseline mean and standard deviation for both groups was set based on the study of Winter et al. [22]. Based on these calculations, a total of 34 participants (17 to each group) would be needed to provide 80% power with a 5% significance level. As the survival rate is over 80%, we increased the target enrollment by 20%, resulting in a sample size of 40 patients. The treatment effect was set to 20% difference between groups targeting 360 gait cycles per day mean difference (assuming SD of 360 for the mean change) in the change between groups.

### Randomization and blinding

Participants are randomly assigned into the intervention and control groups. The randomization is conducted by a computer-generated list based on block randomization with randomly selected block sizes of 2 to 4. In the beginning, the participants do not know whether they are in a control or intervention group, although group contamination is possible as patients may be treated in the same unit making the blinding to the group allocation impossible. However, the information given before obtaining the informed consent is only exposing the aim of researching two different practices of advice and the outcome measurements. Blinding the researcher is not possible either because the researcher is personally giving the participants the intervention guidance. However, the researcher is not conducting the motor performance tests, so the risk for the researcher’s bias affecting the results is minor. The physical therapists that conduct the motor performance assessments are blinded to the group allocation and they do not know in which group the child is allocated unless the children reveal it by themselves.

### Adverse events

Serious adverse events due to the studied intervention are not expected. However, the adverse events may be difficult to recognize and distinguish from the treatment side effects. Active video gaming may cause light-adverse events such as muscle and joint ache and tiring eyes. Nintendo Wii™ health and safety precautions warn that “anyone who has had a seizure, loss of awareness, or other symptoms linked to an epileptic condition” should consult a physician before playing these games [29]. To ensure safety in this study, the treating oncologist checks the eligibility criteria and gives his/her approval for the patients to participate.

### Data analysis

The quantitative data from the activity and fatigue questionnaires, M-ABC scores, accelerometers and metabolic risk factors are to be analyzed and tested using statistical methods. Normally-distributed data will be tested for differences between the intervention and control groups by using a two-sample *t*-test and non-normally-distributed data Wilcoxon rank-sum test. Categorical variables will be tested with a chi-squared test. Multivariable analysis will be conducted with potential confounders if the researcher and statistician find it relevant. Statistical software SPSS (IBM SPSS Statistics 21) will be used. The statistical significance will be set at p <0.05.

The activity diaries will be analyzed by counting the continuous length of sedentary periods and the amount of sedentary periods lasting longer than two hours. The activity periods and their lengths will be counted and summarized. These results will be assimilated into the data from the accelerometers and compared between the groups. Activity data will also be surveyed longitudinally over the whole 2.5-year treatment time and reported as descriptive summaries. Game diaries will be analyzed by counting the duration and frequency for the gaming and the games the participants have played the most. The qualitative data from the interviews will be analyzed with content analysis.

The results will be reported following the methodological guidelines for clinical trials outlined in the CONSORT statement [42].

### Research ethics

The study protocol received the support of the Joint Commission on Ethics of Hospital district of Southwest Finland (15.5.2012 § 153). The research approvals were obtained from all participating institutions (24.9.2012 K66/12 No 13059 and 21.3.2013 65 § R13030).

After the treating oncologist has verified the eligible criteria for the study, the researcher meets the families and provides them with information about the study. Families are adequately informed before consent is asked from them, and they have time to consider their participation overnight or for longer if needed. They have an opportunity to ask questions before giving their consent. Written informed consent is asked from all participants over 6 years old and oral acceptance from children under 6 years old. Written informed consent is asked from the caregivers of children under 15 years old. The caregivers of children over 15 years old are informed about the child’s decision to participate. According to the Medical Research Act, participation in the study can be withdrawn or cancelled at any time without the need to justify the decision [44].

Publishing the protocol is part of the ethical deliberation, since medical research on vulnerable groups, such as children with cancer, needs to be planned carefully [62].

## Discussion

Physical activity and exercise interventions in children with cancer have shown to be mostly beneficial [19,27,28,63]. Studies concerning active video games have shown that playing these games may have a positive impact on children’s physical activity, energy expenditure and motivation to exercise [31,32]. Furthermore, these games may also be beneficial for rehabilitation purposes for children with limited physical function [30]. However, the evidence in the field is still limited.

The primary aim of this study is to provide knowledge of the use of active video games with regard to the promotion of physical activity in children with cancer during cancer treatment, both in hospital and at home. We are also examining the intervention’s effect on motor performance, fatigue and metabolic risk factors. Furthermore, highlighting the children’s views about activation and motivation methods they have or would have enjoyed is a new dimension for discussions in this field.

The results will provide information about whether playing active video games has a positive effect on the studied health outcomes during cancer treatment in children and whether playing these games promotes the children’s physical activity. The study is providing information about children’s physical activity, motor skills, fatigue and metabolic risk factors. Through this study, it may be possible to promote children’s rehabilitation, wellbeing and quality of life. Thus, this information can be used in planning children’s cancer treatment procedures and rehabilitation. The ultimate purpose of this study is preventive—by motivating and activating children to be physically active, independently, during their treatment may potentially help survivors to adopt a positive attitude towards physical activity and possibly avoid other health risks later in life.

From an ethical point of view, the study procedures do not encumber participants excessively. The most intense setup is for the first week of the intervention when the activity diary is completed and the accelerometer worn. Nevertheless, filling out the diary may also be therapeutic in a hospital setting, where time might feel going slowly.

Some limitations of this study must be mentioned. Participation in the intervention study may cause the Hawthorne effect, leading to unusual behavior and biased results. We have not found a validation study for the Fitbit accelerometer and it has not yet been used widely for research purposes, which weakens the validity of the results. In addition, we recognize that including the accelerometer measure only twice during the study for each participant, according to the existing resources, weakens the study design. However, we have several other instruments to measure the primary outcome to reduce this possible limitation. Furthermore, the study runs over a long time period, which may have the effect of producing dropouts. In addition, the control group is also wearing the accelerometer and this may affect the behavior of the children, as they may become more motivated to be active.

This study also has several strengths. From a methodological point of view, the study protocol is built to provide diverse and reliable information from the phenomenon of interest. Randomization and blinding the physical therapists strengthens the study design. The follow-up throughout cancer treatment makes it possible to achieve valuable knowledge of the children’s activity in the long term.

### Trial status

The trial started recruitment in January 2013 and is estimated to complete its follow-up assessments in 2016.

## Competing interests

There are no financial or other competing interest to declare.

## Authors’ contributions

LK, LJ, PL, MA, AA and SS participated in the design of the study. The original manuscript was drafted by LK and reviewed and commented by LJ, PL, AA, OJH, JL and SS. JL and OJH helped in choosing the methods for physical activity measurements. TV conducted the statistical analyses. All authors have read and approved the final manuscript.

## Pre-publication history

The pre-publication history for this paper can be accessed here:

http://www.biomedcentral.com/1471-2431/14/94/prepub
